# Thromboelastometry and Platelet Function during Acclimatization to High Altitude

**DOI:** 10.1160/TH17-02-0138

**Published:** 2018-01-05

**Authors:** Alistair S. Rocke, Gordon G. Paterson, Matthew T. Barber, Alexander I. R. Jackson, Shona Main, Calum Stannett, Martin F. Schnopp, J. Kenneth Baillie, Elizabeth H. Horne, Carl Moores, Paul Harrison, Alastair F. Nimmo, A. A. Roger Thompson

**Affiliations:** 1 Apex (Altitude Physiology Expeditions), Edinburgh, United Kingdom; 2 Edinburgh Medical School, University of Edinburgh, Edinburgh, United Kingdom; 3 Clinical and Experimental Sciences Academic Unit, University of Southampton, Southampton, United Kingdom; 4 Division of Genetics and Genomics, The Roslin Institute, University of Edinburgh, Edinburgh, United Kingdom; 5 Department of Clinical Haematology, St James's University Hospital, Leeds Teaching Hospitals NHS Trust, Leeds, United Kingdom; 6 Department of Anaesthesia, Critical Care and Pain Medicine, Royal Infirmary of Edinburgh, NHS Lothian, Edinburgh, United Kingdom; 7 Institute of Inflammation and Ageing, College of Medical and Dental Sciences, University of Birmingham, Birmingham, United Kingdom; 8 Department of Infection, Immunity and Cardiovascular Disease, University of Sheffield, Sheffield, United Kingdom

**Keywords:** hypoxia, high altitude, haemostasis, platelet function, thromboelastometry

## Abstract

Interaction between hypoxia and coagulation is important given the increased risk of thrombotic diseases in chronically hypoxic patients who reside at sea level and in residents at high altitude. Hypoxia alters the proteome of platelets favouring a prothrombotic phenotype, but studies of activation and consumption of specific coagulation factors in hypoxic humans have yielded conflicting results. We tested blood from 63 healthy lowland volunteers acclimatizing to high altitude (5,200 m) using thromboelastometry and assays of platelet function to examine the effects of hypoxia on haemostasis. Using data from two separate cohorts of patients following identical ascent profiles, we detected a significant delay in clot formation, but increased clot strength by day 7 at 5,200 m. The latter finding may be accounted for by the significant rise in platelet count and fibrinogen concentration that occurred during acclimatization. Platelet function assays revealed evidence of platelet hyper-reactivity, with shortened PFA-100 closure times and increased platelet aggregation in response to adenosine diphosphate. Post-expedition results were consistent with the normalization of coagulation following descent to sea level. These robust findings indicate that hypoxia increases platelet reactivity and, with the exception of the paradoxical delay in thromboelastometry clotting time, suggest a prothrombotic phenotype at altitude. Further work to elucidate the mechanism of platelet activation in hypoxia will be important and could impact upon the management of patients with acute or chronic hypoxic respiratory diseases who are at risk of thrombotic events.

## Introduction

Reports of arterial and venous thrombosis at high altitude have fuelled research testing the hypothesis that hypoxia provokes a prothrombotic phenotype. 1 2 3 4 Epidemiological data suggest a 30-fold increased risk of stroke or venous thrombosis following prolonged sojourn at altitudes greater than 3,000 m. 5 Furthermore, individuals from a high altitude area (> 3,000 m) who were admitted to hospital had a stroke incidence of 13.7/1,000 versus 1.05/1,000 in the wider population and notably almost all of these events occurred in patients younger than 45 years who lacked other cardiovascular risk factors. 6 More controversially, hypoxaemia has been proposed as a significant factor in the increased risk of thrombosis in air travellers, as the cabin pressure in most aircraft is equivalent to an altitude of 2,000 to 2,500 m. 7 8 The risk of a venous thromboembolism approximately doubles following a long-haul flight (>4 hours), the so-called economy class syndrome, 9 but studies investigating whether the degree of hypoxia experienced in an aircraft cabin alters markers of activated coagulation pathways have generated inconsistent results. 10 11 12 13 Importantly, the evidence of interplay between hypoxia and coagulation extends to patients who reside at sea level. Diseases characterized by chronic hypoxaemia carry an increased risk of arterial and venous thrombosis. 14 15 16 Therefore, at both high and low altitudes, greater understanding of the interaction between hypoxia and haemostasis is important and may inform patient management strategies.

Field studies investigating coagulation at altitude are inherently difficult. Cold, exercise, heterogeneous ascent profiles, and sensitivity of equipment and assays potentially compromise conclusions. We aimed to reduce these problems by conducting near-patient tests in a high-altitude laboratory setting after a controlled non-exertional ascent. In two separate groups of patients, we investigated the effects of hypobaric hypoxia on thromboelastometry and platelet function, testing the hypothesis that hypoxia activates coagulation.

## Methods

Data are included from two expeditions, Apex 2 and Apex 4. Both studies were approved by the Lothian Research Ethics Committee and all participants gave written informed consent. Participants received no remuneration for their role in the research.

### Ascent Profile and Sample Collection

**Fig. 1:**
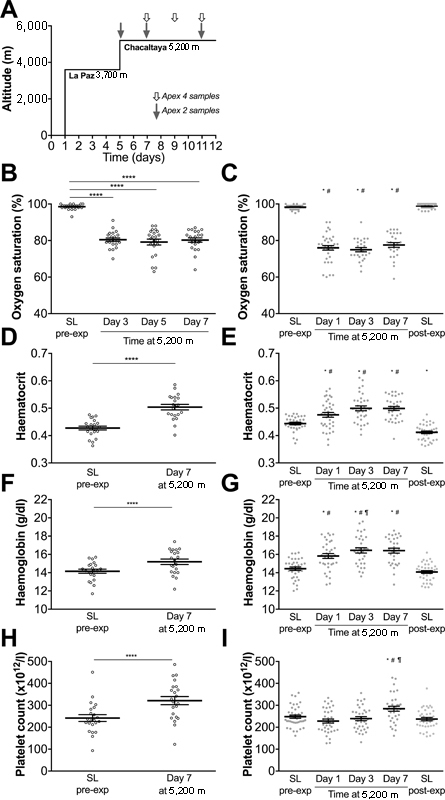
Oxygen saturation, haematocrit, haemoglobin concentration and platelet count on two expeditions (Apex 2 and Apex 4) to 5,200 m. ( **A** ) Patients on both expeditions were exposed to an identical ascent profile. Timing of blood samples at altitude are indicated by shaded arrows (Apex 2) and open arrows (Apex 4). ( **B, C** ) Peripheral oxygen saturations measured at rest on Apex 2 ( **C** ) and Apex 4 ( **B** ). ( **D–I** ) Blood was collected at the time points indicated on Apex 4 ( **D, F, H** ) and Apex 2 ( **E, G, I** ), and haematocrit ( **D, E** ), haemoglobin concentration ( **F, G** ) and platelet count ( **H, I** ) were analysed using automated clinical analysers. Data are mean (horizontal bars) ± SEM. SL, sea level. Apex 2 data points are represented by closed circles; Apex 4 data points are represented by open circles. ( **B, D, F, H** ) **** *p* < 0.0001 using paired *t* -tests. ( **C, E, G, I** ) * *p* < 0.005 versus SL pre-expedition, ^#^ *p* < 0.005 versus SL post-expedition, ^¶^ *p* < 0.005 versus day 1 at 5,200 m using paired *t* -tests corrected for multiple comparisons using Bonferroni method, *p* < 0.005 accepted as significant. ( **B** ) Significance level of *p* < 0.0083 or ( **D, F, H** ) *p* < 0.05 accepted as significant.

All patients were resident at altitudes less than 600 m and had not been to heights greater than 1,500 m in the 3 months preceding the study. Patients flew to La Paz, Bolivia (3,600 m), and spent 4 or 5 days there before ascending for 90 minutes by road to the Chacaltaya Laboratory (5,200 m). Fig. 1A shows the ascent profile of the expeditions and the timing of blood sampling. Sea-level samples were taken before the expedition on Apex 4 but after the expedition on Apex 2. Blood was drawn using a 21-gauge needle, with minimal tourniquet pressure and collected in sterile tubes (Sarstedt, Nümbrecht, Germany) containing sodium citrate or EDTA. Peripheral arterial oxygen saturation (SpO _2_ ) was measured for each patient prior to departure and on each day of investigation using a pulse oximeter (Masimo Rad 5, Irvine, United States). Patients self-recorded symptoms of acute mountain sickness (AMS) using the Lake Louise self-assessment score (LLS) 17 and a visual analogue scale symptom score. 18

### Thromboelastometry Analysis

Thromboelastometry was performed on Apex 2 using a ROTEG05 (Pentapharm GmbH, Munich, Germany) and on Apex 4 using a ROTEM Pactem analyser (TEM International GmbH, Munich, Germany). Each citrated blood sample was assayed within 60 minutes of venepuncture using activators of the extrinsic (EXTEM: tissue factor) and intrinsic (INTEM: phospholipid and ellagic acid) pathways and results were recorded digitally. FIBTEM (cytochalasin D and tissue factor) tests were performed on each blood sample on Apex 4. Internal quality control tests were also recorded at regular intervals throughout the sampling period and values fell within the accepted ranges at all altitudes. Each rotational thromboelastometry (ROTEM) test requires 300 µL of citrated whole blood. Tests were run to obtain an A20 clot amplitude at 20 minutes, and results of clotting time (CT) and clot formation time (CFT, the time from the start of clot formation to thromboelastometry trace amplitude of 20 mm) were recorded.

### Platelet Function Analysis

On Apex 2, citrated blood was analysed, within 5 minutes, by a PFA-100 analyser (Siemens Diagnostics, supplied by Sysmex UK Ltd.). PFA-100 closure time was recorded using collagen-epinephrine cartridges. On Apex 4, blood was taken into a Hirudin tube (Roche Diagnostics, UK) and Multiplate analysis was conducted exactly 20 minutes following venepuncture. Platelet activation with thrombin receptor activating peptide (TRAP, 32 μM), collagen (3.2 μg/mL) and adenosine diphosphate (ADP, 20 μM) stimulation was performed and quantified using the area under the curve of arbitrary aggregation units against time (AUC).

### Full Blood Count Analysis

Full blood count (FBC) analysis was performed at sea level using a KX21-N (Sysmex, UK) within 24 hours of blood collection in EDTA. FBC was measured in La Paz, Bolivia, using a Micros 60 (Horiba, Northampton, UK) 30 hours after blood collection at 5,200 m. The EDTA tubes had been stored and transported upright at 20°C.

### Plasma Preparation

Citrated venous blood (10 mL) was centrifuged immediately at 2,500 g for 15 minutes and the supernatant pipetted into a 10-mL propylene centrifugation tube. Plasma was centrifuged again at 2,500 g for 15 minutes (Labofuge 200, Kendro Laboratory Services, Herts, UK). Plasma aliquots of 500 µL were pipetted into 1 mL propylene aliquot tubes, stored vertically, snap frozen to −80°C and transported on dry ice by specialist international courier to a −80°C storage facility in the UK. Plasma fibrinogen, prothrombin time (PT) and activated partial thromboplastin time (aPTT) were measured in a NHS haematology laboratory.

### Statistical Analysis

Statistical analysis was performed using GraphPad Prism 6.0. The normality of datasets was assessed using Kolmogorov–Smirnov tests and paired *t* -tests were used to compare differences in parameters between sample days. Comparisons of sea-level results versus each time point at altitude and between time points at altitude were made (e.g. for measures recorded on four occasions, six comparisons were made) and *p* -value was adjusted by the Bonferroni method. Therefore, for measures recorded on four occasions, *p* < 0.0083 (0.05/6) was accepted as significant. Relationships between continuous variables were examined by means of individual scatter-plots and where these graphs were suggestive of at least a very weak linear trend, Pearson's correlation coefficients were calculated and R-squared provided as a measure of the strength of the corresponding linear trends.

## Results

Data are included from 63 patients across two expeditions and comparative demographics are presented in Table 1 . On Apex 2, seven patients were evacuated from the laboratory at 5,200 m with symptoms of AMS after the first altitude sample had been collected. Data were included until evacuation. No patients were on medication to treat AMS, but they were permitted to take paracetamol. A further 13 measurements were lost in different patients on different test days (10 on Apex 2, 3 on Apex 4) due to logistical or technical problems.

**Table 1:** Comparative demographic data of the study populations on Apex 2 and Apex 4 expeditions, including prevalence of AMS at 5,200 m

	Apex 2	Apex 4
Number of patients	41	22
Male/Female, %	61/39	54/46
Mean BMI pre-expedition (SD)	22.87 (2.67)	22.31 (2.82)
Median age (range)	21 (19–30)	19 (18–27)
Prevalence AMS		
Day 1	65%	—
Day 3	54%	46%
Day 5	21%	33%
Day 7	13%	21%
Median LLS (range)		
Day 1	4 (0–10)	—
Day 3	3 (0–13)	3 (0–7)
Day 5	1 (0–8)	2 (0–8)
Day 7	1 (0–6)	1 (0–9)

Abbreviations: AMS, acute mountain sickness; BMI, body mass index (kg/m ^2^ ); LLS, Lake Louise score; SD, standard deviation.

Profound hypoxaemia was observed from day 1 at 5,200 m and persisted for the duration of the sojourn at altitude ( Fig. 1B, C ). Haematocrit rose significantly after 4 days of acclimatization at 3,600 m and rose further during the sojourn at 5,200 m ( Fig. 1D, E ), with a corresponding increase in haemoglobin concentration ( Fig. 1F, G ). Platelet count rose by day 7 at 5,200 m on both expeditions ( Fig. 1H, I ). Haematocrit, haemoglobin concentration and platelet count had returned to normal by the time Apex 2 post-expedition sea-level samples were obtained ( Figs. 1E, G, I ).

**Fig. 2:**
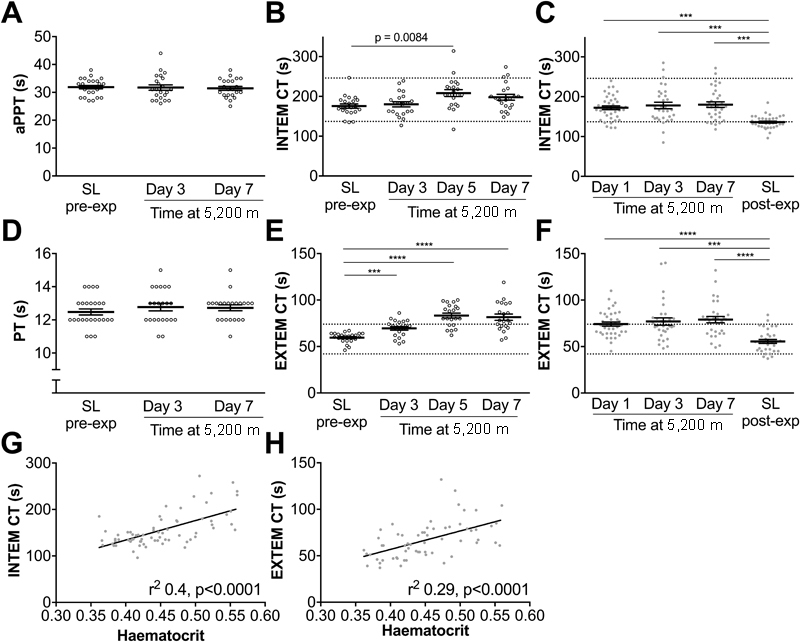
Hypobaric hypoxia prolongs clotting time (CT) assessed by thromboelastometry. ( **A, D** ) aPTT and PT were measured using citrated plasma collected on Apex 4. Whole blood was analysed using thromboelastometry on Apex 4 ( **B, E** ) and Apex 2 ( **C, F** ) using the coagulation activators, ellagic acid (INTEM)( **B, C** ) or tissue factor (EXTEM)( **E, F** ). CT is the time from the start of the assay until the thromboelastometry trace reaches an amplitude of 2 mm, considered to be the start of clot formation. Data are mean (horizontal bars) ± SEM. ( **A–F** ) *** *p* < 0.001, **** *p* < 0.0001 using paired *t* -tests corrected for multiple comparisons using the Bonferroni method, *p* < 0.0083 accepted as significant. ( **G, H** ) Correlations between haematocrit (pooling values from sea level and day 7 at 5,200 m) and INTEM CT ( **G** ) and EXTEM CT ( **H** ) on Apex 2. Apex 2 data points are represented by closed circles, Apex 4 data points are represented by open circles. Reference ranges for thromboelastometry parameters are provided as dashed lines on each graph. 51

The traditional measures of coagulation, aPTT and PT were not significantly different compared with sea-level values ( Fig. 2A, D ). However, thromboelastometry demonstrated that the initiation of clotting (CT) was delayed at altitude. The delay occurred upon activation of blood with either ellagic acid (INTEM; Fig. 2C ) or tissue factor (EXTEM; Fig. 2E, F ), although on the Apex 4 expedition, the increase in INTEM did not reach statistical significance ( Fig. 2B ). CT was significantly prolonged compared with the post-expedition sea-level values at all time points on Apex 2 ( Fig. 2C, F ). Modest but significant correlations were observed between haematocrit (pooling values from sea level and day 7 at 5,200 m) and both INTEM CT ( *r* ^2^ 0.4, *p* < 0.0001) and EXTEM CT ( *r* ^2^ 0.29, *p* < 0.0001) on Apex 2 ( Fig. 2G, H ) and Apex 4 (haematocrit vs. INTEM CT ( *r* ^2^ 0.17, *p* = 0.007) or versus EXTEM CT ( *r* ^2^ 0.41, *p* < 0.0001; Suppl 1A , B ). No change in CFT was observed on either expedition following intrinsic (INTEM) activation ( Suppl 2A , B ). While there was a significant reduction in CFT following tissue factor (EXTEM) activation on days 1 and 7 on Apex 2 ( Suppl 2D ), this was not mirrored by the Apex 4 data ( Suppl 2C ).

**Fig. 3:**
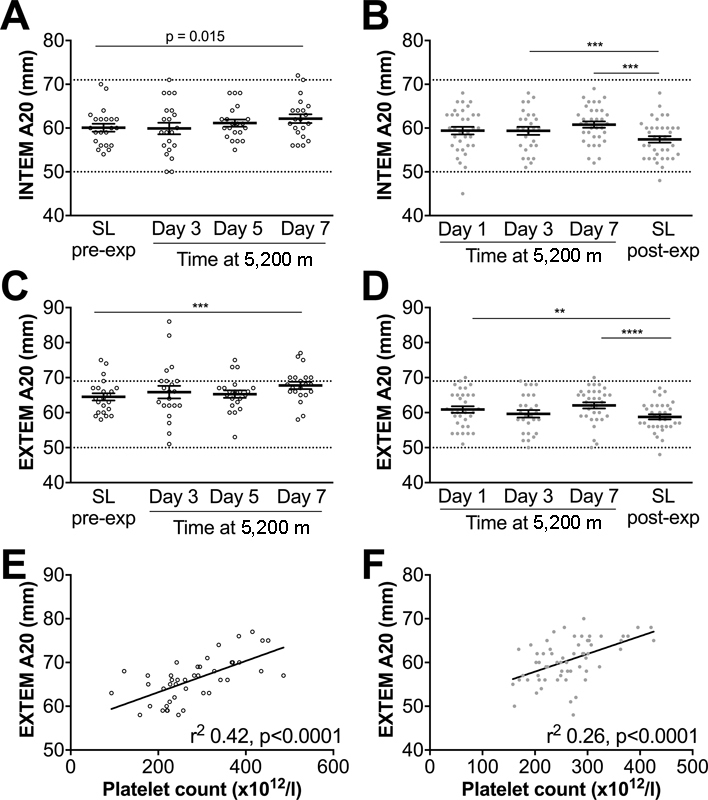
Thromboelastometry measurements demonstrate an increase in clot strength at 5,200 m. Whole blood was analysed using thromboelastometry on Apex 4 ( **A, C** ) and Apex 2 ( **B, D** ) using INTEM ( **A, B** ) or EXTEM ( **C, D** ). A20 is the trace amplitude after 20 minutes and is a measure of clot strength. Data are mean (horizontal bars) ± SEM. ( **A-D** ) *** *p* < 0.001, **** *p* < 0.0001 using paired *t* -tests corrected for multiple comparisons using the Bonferroni method, *p* < 0.0083 accepted as significant. ( **E, F** ) Correlations between platelet count (pooling values from sea level and day 7 at 5,200 m) and EXTEM A20 on Apex 2 ( **E** ) and Apex 4 ( **F** ). Apex 2 data points are represented by closed circles; Apex 4 data points are represented by open circles. Reference ranges for thromboelastometry parameters are provided as dashed lines on each graph. 51

EXTEM trace amplitude at 20 minutes (A20) was significantly greater on day 7 at 5,200 m on both expeditions ( Fig. 3C, D ) and INTEM A20 was greater by day 3 at 5,200 m on Apex 2 ( Fig. 3B ). The increase in INTEM A20 on Apex 4 did not reach statistical significance ( Fig. 3A ). Platelet count is an important determinant of clot strength and we detected significant, although weak, correlations between platelet count and EXTEM A20 on both expeditions ( Fig. 3E, F ).

**Fig. 4:**
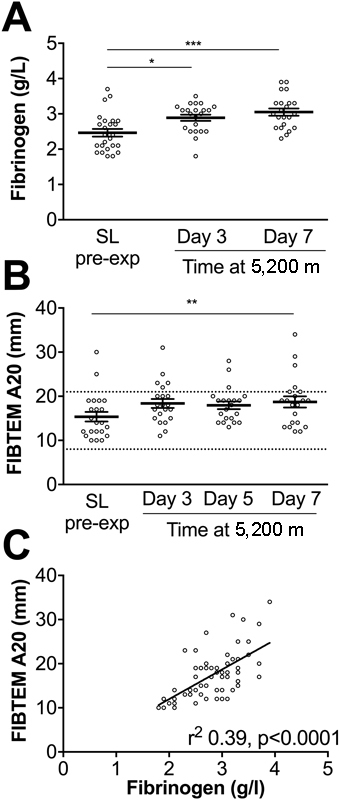
Increased fibrinogen and fibrin-dependent clot strength at altitude. ( **A** ) Fibrinogen levels were measured on Apex 4 at the time points indicated. ( **B** ) Whole blood thromboelastometry analysis of A20 using FIBTEM on Apex 4. Data are mean (horizontal bars) ± SEM. * *p* < 0.05, ** *p* < 0.01, *** *p* < 0.001 using paired *t* -tests corrected for multiple comparisons using the Bonferroni method, (A) *p* < 0.016 or ( **B** ) *p* < 0.0083 accepted as significant. ( **C** ) Correlation between plasma fibrinogen concentration and FIBTEM A20 (pooling values from sea level and day 7 at 5,200 m). Reference ranges for thromboelastometry parameters are provided as dashed lines on each graph. 51

FIBTEM uses cytochalasin D to inhibit platelet function and assesses fibrin clot strength in the absence of platelet activation. Fibrinogen levels were significantly elevated on day 3 and day 7 at 5,200 m compared with sea level ( Fig. 4A ). Consistent with this finding, FIBTEM A20 was significantly increased by day 7 ( Fig. 4B ) and fibrinogen levels correlated with FIBTEM A20 ( Fig. 4C ).

**Fig. 5:**
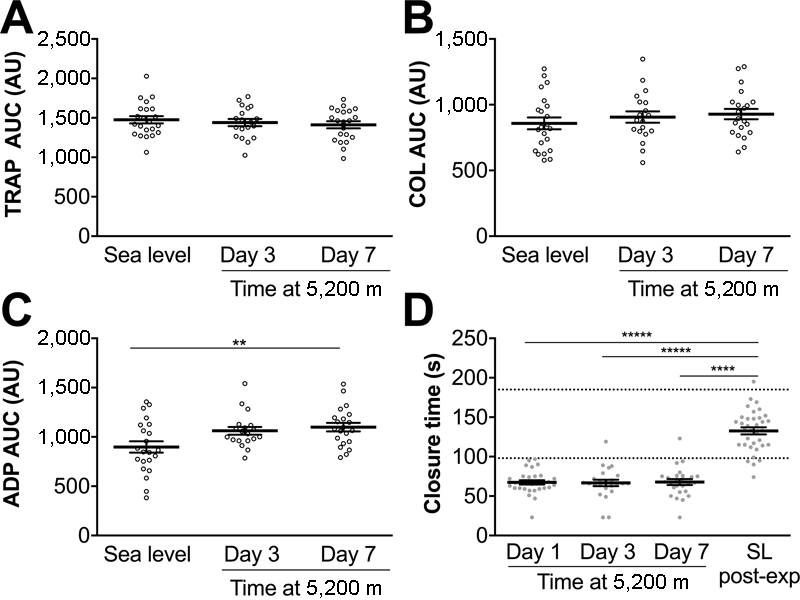
Evidence of increased platelet reactivity at 5,200 m. Platelet function was assessed on Apex 4 using Multiplate electrode aggregometry assays on whole blood activated by ( **A** ) thrombin receptor activating peptide (TRAP), ( **B** ) Collagen (COL) or (C) adenosine diphosphate (ADP). Area under the curve (AUC) represents the degree of platelet aggregation over time. ( **D** ) Platelet function was assessed on Apex 2 using a PFA-100 device and collagen-epinephrine cartridges. Increased platelet reactivity reduces closure time. Data are mean (horizontal bars) ± SEM. ** *p* < 0.01, ***** *p* < 0.00001 using paired *t* -tests corrected for multiple comparisons using the Bonferroni method, ( **C** ) *p* < 0.016 or ( **D** ) *p* < 0.0083 accepted as significant. Apex 2 data points are represented by closed circles; Apex 4 data points are represented by open circles.

Multiplate assays demonstrated no significant changes in platelet aggregation upon stimulation with either TRAP or collagen ( Fig. 5A, B ). Aggregation in response to ADP was significantly greater on day 7 at 5,200 m ( Fig. 5C ). As a further indication of platelet reactivity, PFA-100 closure times, using collagen-epinephrine cartridges, were profoundly reduced from day 1 at 5,200 m and remained low during the 7-day altitude sojourn, compared with post-expedition sea-level values and published reference ranges ( Fig. 5D ). 19

## Discussion

Hypoxia, whether local or systemic, frequently complicates thromboembolism, but the role it plays in altering haemostasis is poorly understood. Using near-patient tests on healthy volunteers in an extreme hypoxic environment, we have gathered unique data describing altitude-induced alterations in coagulation. Data from two separate expeditions to high altitude revealed an increase in the clotting time at 5,200 m when compared with pre-expedition or post-expedition sea-level values, as measured by thromboelastometry. Although there was a latency in clot formation, we found evidence of an increase in clot strength, indicated by an increase in amplitude of the TEM trace 20 minutes after clot was first detected. Furthermore, after 3 days at altitude, there was an increase in plasma fibrinogen concentration and this was associated with an increase in fibrin-formed clot strength. Finally, using two different measurements of platelet function, we revealed an increase in platelet reactivity at high altitude in response to ADP and collagen-epinephrine stimulation.

Rotational thromboelastometry allows a measurement of global haemostatic function in whole blood samples. The technique analyses the dynamics of clot formation and gives information on the interaction of platelets, clotting factors and fibrinogen to generate a graphical depiction of the coagulation process. Unlike specific blood component assays, thromboelastometry examines the complete interaction between plasma constituents revealing the net consequences of abnormalities affecting the formation of the clot. Despite our apparent paradoxical findings of a latency in clot formation but an increase in clot strength, our results are remarkably consistent with previous work performed at altitude. Using a similar method, thromboelastography (TEG), the Caudwell Everest expedition assessed coagulation at 5,300 m in 17 healthy volunteers who ascended to the base camp of Mount Everest over 13 days. 20 Reaction (R) time and coagulation (K) time (comparable to CT and CFT, respectively) were both increased compared with sea level on arrival at 5,300 m, and the α-angle was decreased. 20 Modesti et al conducted a similar study using ROTEM at Everest base camp (5,300 m) with 47 volunteers and discovered increases in CT, CFT and flattened α-angle using an INTEM assay. 21 Consistent with our findings, a significant increase in MCF was observed after a 10-day sojourn at Everest base camp. 21 Interestingly, significantly shorter CFT and higher MCF values were found in patients who developed AMS during that expedition compared with those who did not. 21 Although the study by Modesti et al was conducted at a similar altitude to our study, it should be noted that the ascent profile in our study was more rapid. These differences could explain why we did not observe any association between TEM parameters and AMS (data not shown).

The prolongation of CT described here and by others 20 21 suggests a delay in clot formation at altitude. Prolonged TEM CT is described in the context of clotting factor deficiency. 22 23 However, studies have shown either no change or increases in the level of plasma clotting factors at altitudes of greater than 4,500 m. 24 25 Martin et al proposed that the altitude-induced prolongation of TEG, R time, might involve the inhibition of clotting factor activity by nitric oxide (NO). 20 There is evidence of increased NO formation in lowlanders ascending to high altitude, 26 while ex vivo administration of nitrite (a NO precursor) to hypoxic blood from healthy volunteers increased R time. 27 However, NO has also been shown to reduce clot strength, 27 28 a finding inconsistent with our results. Changes in blood pH are also known to alter CT, but while acidification of blood was previously demonstrated to prolong CT, induced alkalosis had no effect. 29 Therefore, the hypocapnic alkalosis that occurs on ascent to altitude would not explain our findings. Interestingly, a similar delay in coagulation has been reported in patients with congenital cyanotic heart disease (CCHD), a population with hypoxaemia and high haematocrit. 30 A study of 75 patients with CCHD showed a correlation between haematocrit and R time assessed by TEG, 30 a finding mirrored by the modest but significant correlations between haematocrit and CT results on both Apex expeditions. Nonetheless, it is possible that the observed changes in TEM CT are due to a relative dilution of coagulation factors within the whole blood sample, caused by the rise in haematocrit. This phenomenon has been simulated by ex vivo addition of autologous red cells to elevate the haematocrit of blood from healthy volunteers 31 and converse effects are observed in patients with anemia. 32 Moreover, it is notable that we did not find changes in conventional clinical measurements of coagulation (PT and aPTT) performed on citrated plasma and therefore independent of haematocrit. Experimental alteration of haematocrit at sea level and at high altitude would be necessary to confirm whether the observed changes in clot latency were due to hypoxia or haematocrit.

In contrast to the results from patients with CCHD, 30 31 we observed a rise in clot strength as assessed by TEM trace amplitude after 20 minutes. Indeed, elevated haematocrit was associated with reduced maximum clot firmness (MCF) assessed by EXTEM or FIBTEM in CCHD patients or following ex vivo manipulation of haematocrit levels. 31 Other factors contributing to clot strength assessed by TEG include fibrinogen levels and platelet count 33 34 and it is possible that differences in these parameters explain why clot strength increased in our study despite the rise in haematocrit. Notably, patients with CCHD tend to have lower platelet counts than healthy volunteers 31 and we observed increases in both platelet count and fibrinogen in our patients over time at 5,200 m. Furthermore, we detected significant correlations between platelet count and EXTEM A20 on both expeditions. The addition of FIBTEM and fibrinogen measurements on the Apex 4 expedition allowed us to determine whether platelet count alone accounted for the increased A20, as cytochalasin D eliminates the platelet component of clot strength. However, A20 was also elevated when assessed by FIBTEM on the Apex 4 expedition and therefore the increase in EXTEM A20 is likely to be due to a combination of increases in platelet count and plasma fibrinogen concentration.

Our finding of increased platelet count at altitude on both expeditions is notable given inconsistent historical results. 35 36 The rise in platelet count lagged temporally behind that of haematocrit, suggesting that it was not simply a consequence of haemoconcentration. The potential importance of platelet count in the context of hypoxaemia is indicated by the independent association between higher platelet counts and increased mortality in patients with lung disease who reside at sea level. 37 In addition, we found evidence of increased platelet reactivity at altitude. PFA-100 closure time was significantly shorter than post-expedition sea-level results and at or below the lower limit of published reference ranges from day 1 at 5,200 m and remained low for the duration of the altitude sojourn. Our results are consistent with a study investigating platelet function in 24 patients known to be susceptible to high altitude pulmonary oedema and 10 healthy controls. 38 Lehmann et al described a reduction in collagen-epinephrine closure times of 27% on arrival at 4,559 m after a 2-day ascent and found no difference in closure times between HAPE-susceptible patients and controls. 38 Interestingly, the study by Lehmann et al found a significant fall in platelet count on arrival at altitude and attributed this to platelet aggregation and consumption secondary to hypoxia-induced activation. A key difference between our study and that of Lehmann et al was the nonexertional ascent on Apex expeditions, compared with several hours of hiking. While it is possible that exercise may have influenced platelet numbers, the similarities in platelet reactivity suggest that the observed differences in platelet counts are not due to aggregation. However, we cannot exclude the possibility of a transient, unmeasured, drop in platelet count in our patients during the initial ascent to 3,600 m on arrival in La Paz.

To explore how hypoxia alters platelet reactivity, we performed further tests of platelet activation on Apex 4, screening different platelet agonists using Multiplate impedance aggregometry assays. These revealed a specific increase in platelet aggregation in response to ADP by day 7 at 5,200 m. Consistent with this finding, platelets isolated from rats exposed to hypoxia for 6 hours also display increased aggregation in response to ADP, associated with increased ATP release and increased surface expression of platelet activation markers. 39 Indeed, the study by Tyagi et al demonstrated important hypoxia-induced alterations in the platelet proteome, including induction of platelet-derived tissue factor and fibrinogen and suppression of antithrombotic proteins. 39 Calpain proteases were identified as important mediators of hypoxia-induced platelet hyper-responsiveness and are known to be activated by increased intracellular Ca ^2+^ , 40 a downstream consequence of P2Y _1_ receptor activation by ADP. Although this proposed interaction between ADP and calpains requires further investigation, the alteration of the hypoxic platelet proteome adds credibility to our observed changes in platelet function at altitude and suggests a possible mechanism for the rise in fibrinogen concentration at altitude.

Multiplate results may be affected by platelet count and haematocrit. 41 42 However, suspensions of increasing platelet counts provoked a nonspecific increase in aggregation in response to several agonists rather than a specific response to ADP alone. 41 Furthermore, the early increase in haematocrit ( Fig. 1E ) preceded the significant rise in ADP-induced platelet aggregation, which did not occur until day 7 at 5,200 m. A rise in haematocrit can also reduce the closure time measured by PFA-100. 43 However, we found no correlation between the rise in haematocrit on ascent to altitude and change in closure time (data not shown). In addition, the magnitude of the reduction in closure time exceeds that which would be expected by the observed rise in haematocrit. 44

This work has several limitations. First, while we provide evidence of hypercoagulability in the form of increased TEM clot strength and platelet activation, how these parameters might relate to risk of subsequent thrombotic events is not clear. Studies using TEG have indicated a possible relationship between clot strength and thrombosis risk in the context of surgery, 45 functional outcome following stroke 46 and recurrent ischaemic events after percutaneous coronary intervention. 47 While there is agreement between TEG and ROTEM measures of clot strength, 48 direct extrapolation of the quantified risk from TEG parameters to ROTEM is not possible and there are few published studies investigating links between ROTEM measures of clot strength and thrombotic risk. Hincker et al reported a relationship between preoperative ROTEM parameters and postoperative risk of thromboembolic complications following cardiac surgery. 49 Patients who developed thromboembolic complications had significantly higher preoperative EXTEM MCF values, but notably other parameters that might indicate hypercoagulability were also abnormal (shorter CFT and steeper α-angle). The odds ratio for developing a postoperative complication if EXTEM MCF was outside the normal range was 4.65. However, only 4 of the 30 patients who developed complications fulfilled this criterion, implying that values need not lie outside the normal range. Indeed, it is plausible that there is a more continuous relationship between clot strength and thrombotic risk, but further work using TEM is required to establish such a link. Second, the applicability of our findings to high altitude dwellers or patients with chronic respiratory disease at sea level is also unclear, and studies during longer term hypoxic exposure may have more direct relevance to the increased risk of thrombotic events in such groups. Finally, although our post-expedition data suggest that the assessed coagulation parameters normalize on return to sea level, only assessment of pre-expedition and post-expedition data from the same cohort would provide evidence to confirm this conclusion. Moreover, as it has recently been demonstrated that short-term acclimatization to hypoxia has a prolonged impact on murine leukocyte transcriptional responses and metabolism on reexposure to acute hypoxia, 50 it would also be of interest to collect data after re-ascent to altitude in acclimatized volunteers.

In conclusion, we demonstrate evidence of increased platelet reactivity and increased clot strength in whole blood assays in healthy volunteers exposed to altitude hypoxia. The increase in clot strength may be explained by rises in platelet count and fibrinogen while the recent discovery of hypoxia-induced changes in the platelet proteome 39 may provide insight into our observations of increased platelet reactivity. Whether our results indicate an increased risk of thrombotic events at altitude is not clear and requires further study. Nonetheless, our finding of increased platelet reactivity at high altitude has potential implications for patients who reside at sea level with acute or chronic diseases caused or complicated by hypoxia and known to be at risk of thrombotic events. Understanding the mechanisms driving platelet activation and clot behaviour in hypoxia might impact upon treatment of such patients at sea-level, in addition to increasing our understanding of altitude-related thromboses.

## Notes

What is known about this topic?

- Epidemiological evidence suggests that exposure to high altitude is associated with an increased risk of thrombotic events.
- Published data from assays of blood coagulation have not conclusively supported a prothrombotic phenotype at high altitude.

**What does this paper add?**

- In two separate cohorts of healthy volunteers ascending to high altitude, we report evidence of altered haemostasis.
- We found evidence of increased clot strength, assessed by thromboelastometry, and evidence of increased platelet reactivity after 7 days at 5,200 m.
- Our data imply that hypoxia alters platelet reactivity, a finding of importance to those ascending to high altitude which may also be of relevance to patients who reside at sea level with diseases characterized by acute hypoxia.
