# Supplementary material for: Thromboelastometry and Platelet Function during Acclimatization to High Altitude
Source: Thromb Haemost. 2018 Jan 5;118(1):63–71. doi: 10.1160/TH17-02-0138 (PMC6260116; doi:10.1160/TH17-02-0138)
Supplement: Supplementary file 1 — Supplementary Figures [file 10-1160-th17-02-0138-s170138.pdf]

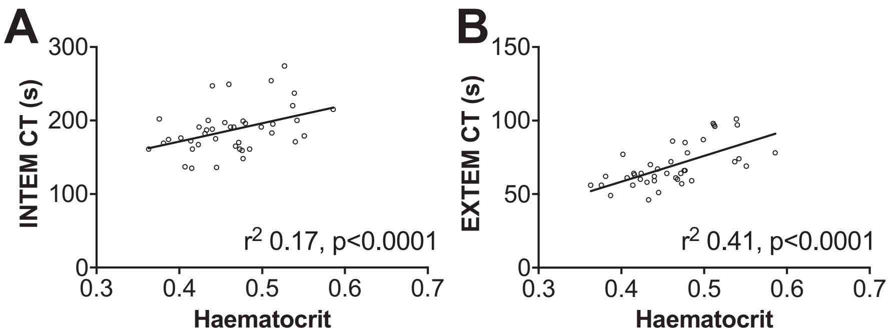

Supplemental Figure 1 Correlations between haematocrit and TEM clotting time (CT). (A, B) Correlations between haematocrit (pooling values from sea level and day 7 at 5200 m) and INTEM CT (A) and EXTEM CT (B) on Apex 4.

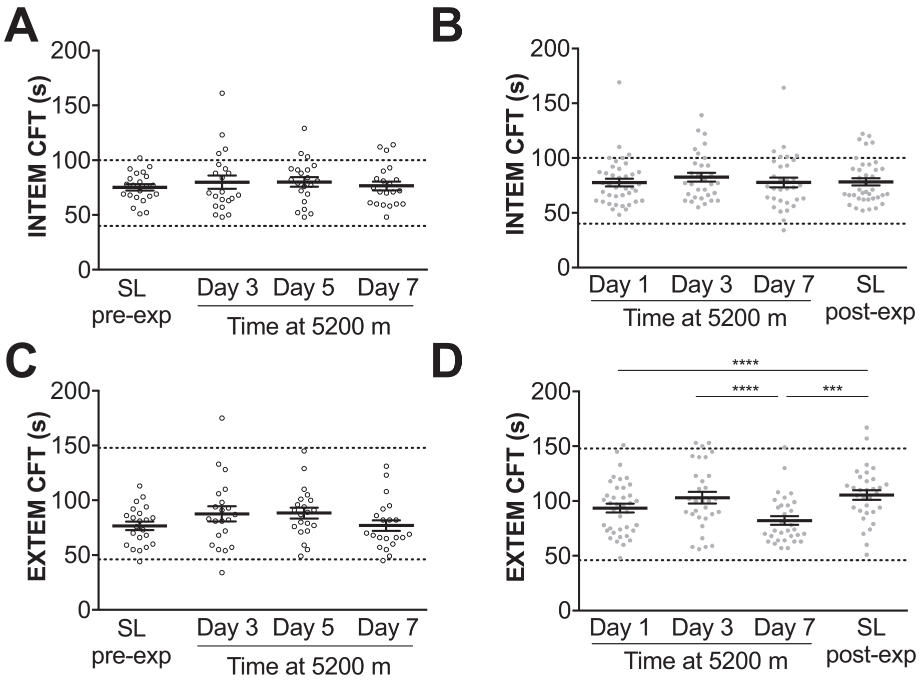

Supplemental Figure 2 Thromboelastometry measurements of clot formation time (CFT). Whole blood was analysed using thromboelastometry on Apex 4 (A, C) and Apex 2 (B, D) using INTEM (A, B) or EXTEM (C, D). Clot formation time (CFT) is the time taken from a thromboelastometry trace amplitude of 2 mm above baseline to 20 mm above baseline. Data are mean (horizontal bars) +/− SEM. $^{*}p < 0.05$ using paired t-tests corrected for multiple comparisons.
